# Supplementary material for: Proteotranscriptomic Profiling of the Toxic Mucus of Kulikovia alborostrata (Pilidiophora, Nemertea)
Source: Toxins (Basel). 2024 Dec 26;17(1):5. doi: 10.3390/toxins17010005 (PMC11768458; doi:10.3390/toxins17010005)
Supplement: Supplementary file 1 [file toxins-17-00005-s001.zip › Supplementary File S4.zip!/ORF 006024 LINALB/230688690e3e92c6/ss_report.pdf]

Email	vas9i-kz@mail.ru
Description	ORF_006024_LINALB
Date	Fri Apr 7 01:53:58 BST 2023
Unique Job ID	230688690e3e92c6

Phyre2

Secondary structure and disorder prediction

	1 . . . . 10 . . . . 20 . . . . 30 . . . . 40 . . .
Sequence	HEMHDFSEEV S V NKRCSKHGRCGNDYDCCPGY V C S RKYKECSR
Secondary structure	
SS confidence	
Disorder	? ?
Disorder confidence	

Confidence Key

High(9) Low (0)

? Disordered (44%)

Alpha helix (0%)

Beta strand (16%)
